# Investigation of Calcium Channel Blockers as Antiprotozoal Agents and Their Interference in the Metabolism of* Leishmania (L.) infantum*

**DOI:** 10.1155/2016/1523691

**Published:** 2016-01-28

**Authors:** Juliana Quero Reimão, Juliana Tonini Mesquita, Daiane Dias Ferreira, Andre Gustavo Tempone

**Affiliations:** ^1^Centre for Parasitology and Mycology, Institute Adolfo Lutz, Secretary of Health of São Paulo State, Avenida Dr. Arnaldo 351, 01246-000 São Paulo, SP, Brazil; ^2^Departamento de Morfologia e Patologia Básica, Faculdade de Medicina de Jundiaí, Rua Francisco Telles 250, 13202-550 Jundiai, SP, Brazil

## Abstract

Leishmaniasis and Chagas disease are neglected parasitic diseases endemic in developing countries; efforts to find new therapies remain a priority. Calcium channel blockers (CCBs) are drugs in clinical use for hypertension and other heart pathologies. Based on previous reports about the antileishmanial activity of dihydropyridine-CCBs, this work aimed to investigate whether the* in vitro *anti-*Leishmania infantum* and anti-*Trypanosoma cruzi *activities of this therapeutic class would be shared by other non-dihydropyridine-CCBs. Except for amrinone, our results demonstrated antiprotozoal activity for fendiline, mibefradil, and lidoflazine, with IC_50_ values in a range between 2 and 16 *μ*M and Selectivity Index between 4 and 10. Fendiline demonstrated depolarization of mitochondrial membrane potential, with increased reactive oxygen species production in amlodipine and fendiline treated* Leishmania*, but without plasma membrane disruption. Finally,* in vitro* combinations of amphotericin B, miltefosine, and pentamidine against* L. infantum *showed in isobolograms an additive interaction when these drugs were combined with fendiline, resulting in overall mean sum of fractional inhibitory concentrations between 0.99 and 1.10. These data demonstrated that non-dihydropyridine-CCBs present antiprotozoal activity and could be useful candidates for future* in vivo* efficacy studies against Leishmaniasis and Chagas' disease.

## 1. Introduction

Leishmaniasis is a neglected infectious disease caused by several different species of protozoan parasites of the genus* Leishmania*. It affects 12 million people in 98 countries and territories and is mainly associated with poverty in developing nations. Current strategies to control this disease are mainly based on chemotherapy. Despite being available for the last 100 years, the chemotherapy is based on the use of few drugs, including the antimonial derivatives. High costs of treatment, elevated toxicity, parenteral administration, and the emergence of resistance are the main drawbacks [1]. Considering the few therapeutic options and lack of interest from private sector, the need for novel drugs is evident [2].

Calcium channel blockers (CCBs) are a class of drugs that act by selective inhibition of calcium influx through cellular membranes. They are among the most widely used drugs in cardiovascular medicine with roles not only in hypertension but also in angina and (for some CCBs) tachyarrhythmias [3]. Although often considered as a single class, CCBs can be subdivided into the following groups depending on chemical structure: dihydropyridines (e.g., nifedipine, nimodipine, and amlodipine), the benzothiazepines (e.g., diltiazem), and phenylalkylamines (e.g., verapamil) [4].

Dihydropyridines have been considered promising antiparasitic hits, especially against protozoan parasites. The* in vivo* oral efficacy of amlodipine and lacidipine has been shown in the treatment of* Leishmania (L.) donovani *infected mice [5]. Additionally, the* in vitro *antiparasitic activity of eight clinically used dihydropyridines (azelnidipine, amlodipine, cilnidipine, lercanidipine, nicardipine, nifedipine, nimodipine, and nitrendipine) was demonstrated against a panel of* Leishmania* species and* Trypanosoma cruzi *[6, 7].

Based on previous reports about the antileishmanial activity of dihydropyridines, this work investigated the antiparasitic potential of other non-dihydropyridine-CCBs. For this, the* in vitro *activity of four non-dihydropyridine agents (amrinone, fendiline, mibefradil, and lidoflazine) was tested against different* Leishmania *species and their cytotoxicity to mammalian cells was evaluated. We also investigated the mechanism of action (MoA) involved with the antileishmanial activity of fendiline and the* in vitro *effect of its combination with antileishmanial standard drugs.

## 2. Material and Methods

### 2.1. Drugs and Chemicals

Dimethyl sulfoxide (DMSO), phosphate buffer saline (PBS), 3-[4,5-dimethylthiazol-2-yl]-2,5-diphenyltetrazolium bromide (MTT, thiazol blue), sodium dodecyl sulfate (SDS), RPMI-1640 medium, and M199 medium were purchased from Sigma (St. Louis, MO, USA). Amrinone, fendiline, lidoflazine, and mibefradil were purchased from Sigma (St. Louis, MO, USA). Pentamidine was from Sideron. Pentavalent antimony (Glucantime®) and amlodipine were kindly donated by Bayer (Brazil). Other analytical reagents were purchased from Sigma unless otherwise stated.

### 2.2. Experimental Animals

Golden hamsters and BALB/c mice were supplied by the animal breeding facility at the Adolfo Lutz Institute of São Paulo. They were maintained in sterilized cages under a controlled environment and received water and food* ad libitum*. Golden hamsters were infected each month with amastigotes from the spleen to maintain the strain of* L. (L.) infantum*. BALB/c mice were used for obtaining peritoneal macrophages. Animal procedures were performed with the approval of the Research Ethics Commission, in agreement with the Guidelines for the Care and Use of Laboratory Animals from the National Academy of Sciences.

### 2.3. Parasites and Macrophages

Promastigotes of* L. (L.) amazonensis* (WHO/BR/00/LT0016),* L. (V.) braziliensis* (MHO/BR/75/M2903), and* L. (L.) infantum *(MHOM/BR/1972/LD) [synonymous with* L. (L.) chagasi*] were maintained in M199 medium supplemented with 10% calf serum and 0.25% hemin at 24°C.* L. (L.) infantum *amastigotes were obtained from the spleen of infected hamster by differential centrifugation at the 60–70th days after infection. Macrophages were collected from the peritoneal cavity of BALB/c mice by washing with RPMI-1640 medium supplemented with 10% fetal calf serum and were maintained at 37°C in a 5% CO_2_ humidified incubator.* Trypanosoma cruzi *trypomastigotes (Y strain) were maintained in LLC-MK2 (ATCC CCL 7) cells using RPMI-1640 medium supplemented with 2% calf serum at 37°C in a 5% CO_2_ humidified incubator.

### 2.4. Determination of the* In Vitro* Antileishmanial Activity

To determine the 50% inhibitory concentration (IC_50_) against* Leishmania* promastigotes, the drugs were dissolved in DMSO and diluted with M199 medium in 96-well microplates, with 100 *μ*M as the highest concentration. Each drug was tested twice at eight concentrations prepared in twofold dilutions. Promastigotes were counted in a Neubauer hemocytometer and seeded at 1 × 10^6^ parasites/well with a final volume of 150 *μ*L. Controls with DMSO and without drugs were performed. Pentamidine was used as a standard drug. The plate was incubated for 24 hours at 24°C and the viability of promastigotes was verified by the MTT assay. Briefly, MTT (5 mg/mL) was dissolved in PBS, sterilized through 0.22 *μ*m membrane and 20 *μ*L/well was added, for 4 hours at 24°C. Promastigotes were incubated without compounds and used as a viability control. Formazan extraction was performed using 10% SDS for 18 hours (80 *μ*L/well) at 24°C, and the optical density was determined in a Multiskan MS (UNISCIENCE) plate reader at 550 nm. The 100% viability was expressed based on the optical density of control promastigotes, after normalization. To determine the IC_50_ value against* L. (L.) infantum *intracellular amastigotes, peritoneal macrophages were collected from the peritoneal cavity of BALB/c as described above, and added to 16-well chamber slides (Lab-Tek-NUNC®) at 5 × 10^4^ cells/well. Plates were incubated at 37°C in a 5% CO_2_ humidified incubator for 24 hours.* L. (L.) infantum *amastigotes extracted from spleens and separated by differential centrifugation were added to macrophages at a ratio of 10 : 1 (amastigotes : macrophage). After 24 hours, extracellular parasites were removed by washing, fresh medium containing the drugs and controls was added, and the cells were incubated at 37°C for a period of 120 hours. Further medium changes with fresh drugs were carried out after 72 hours. At the end of the assay, the slides were stained with Giemsa and observed using light microscopy. Glucantime was used as a standard drug. The IC_50_ was determined by the number of infected macrophages in 400 cells.

### 2.5. Determination of the Antitrypanosomal Activity

To determine the IC_50_ against* T. cruzi *trypomastigotes, drugs were dissolved in DMSO and diluted with RPMI-1460 medium in 96-well microplates, with the highest concentration at 100 *μ*M. Trypomastigotes obtained from LLC-MK2 cultures were counted in a Neubauer hemocytometer and seeded at 1 × 10^6^ parasites/well in 96-well microplates. Test drugs were incubated for 24 hours at 37°C in a 5% CO_2_ humidified incubator and the viability of trypomastigotes was verified by the MTT assay as described above. Benznidazole was used as a standard drug.

### 2.6. Cytotoxicity in Mammalian Cells

LLC-MK2 were seeded at 5 × 10^4^ cells/well in 96-well microplates and incubated with drugs with 200 *μ*M as the highest concentration, for 48 hours at 37°C in a 5% CO_2_ humidified incubator. The viability of the cells was determined by the MTT assay as described above. Control cells were incubated in the presence of DMSO and without drugs. Viability of 100% was expressed based on the optical density of control LLC-MK2 cells, after normalization. The Selectivity Index (SI) was given by the ratio between the cytotoxicity in LLC-MK2 cells and the antiparasitic activity.

### 2.7. Investigation of Mitochondrial Membrane Potential

*L. (L.) infantum *promastigotes were washed with PBS and deposited on a 96-well microplate (2 × 10^6^ cells/well) and incubated with amlodipine or fendiline (20 *μ*M) for 60 minutes at 24°C. Then MitoTracker® Red CM-H_2_XROS (500 nM) was added and the incubation was continued for 40 minutes in the dark. Cells were washed twice with HBSS (Hanks Balanced Salt Solution) and the fluorescence was measured using a fluorimetric microplate reader (FilterMax F5 Multi-Mode Microplate Reader-Molecular Devices) with excitation and emission wavelengths of 540 and 595 nm, respectively [8]. Nitazoxanide (60 *μ*g/mL) was used as a positive control [9] and untreated promastigotes were used as negative control.

### 2.8. Analysis of Reactive Oxygen Species (ROS)

*L. (L.) infantum *promastigotes (2 × 10^6^ cells/well) were washed in HBSS medium and incubated with amlodipine or fendiline (20 *μ*M) for 60 minutes at 24°C. To these cells 2′,7′-dichlorodihydrofluorescein diacetate (H_2_DCf-DA) was added (5 *μ*M) and incubation was prolonged for 15 minutes. Then the fluorescence was measured using a fluorimetric microplate reader (FilterMax F5 Multi-Mode Microplate Reader-Molecular Devices) with excitation and emission wavelengths of 485 and 520 nm, respectively. Nitazoxanide (60 *μ*g/mL) was used as positive control [9] and untreated promastigotes were used as negative control.

### 2.9. Evaluation of the Permeability of the Cell Membrane

*L. (L.) infantum* promastigotes were washed with PBS, deposited on a 96-well microplate (2 × 10^6^ parasites/well) and incubated with SYTOX® Green (1 *μ*M) for 15 minutes at 24°C [10]. Amlodipine and fendiline were added at 20 *μ*M and the fluorescence was measured up to 60 minutes. The fluorescence intensity was determined using a fluorimetric microplate reader (FilterMax F5 Multi-Mode Microplate Reader-Molecular Devices) with excitation and emission wavelengths of 485 and 520 nm, respectively. Triton X-100 (0.1%) was used as positive control and untreated promastigotes were used as negative control.

### 2.10. Determination of Drug Interactions

The interactions between drugs were* in vitro *evaluated by modified isobologram method [11, 12]. Fendiline was* in vitro *combined with amphotericin B, miltefosine, and pentamidine. IC_50_ values of individual drugs were obtained against* L. (L.) infantum *promastigotes as described above. These values were used to determine the maximum concentrations of each drug in the combination assay, assuring the IC_50_ in the fourth point of the serial dilution [11]. The highest concentrations of the solutions were prepared in proportions of 5 : 0, 4 : 1, 3 : 2, 2 : 3, 1 : 4, and 0 : 5 of fendiline and partner drug, respectively, which were serially diluted to the seventh well of the microplate in two intercalated serial dilutions (base 2).

### 2.11. Determination of FIC Index, Isobologram Construction, and Classification of the Nature of Interaction

Fifty and ninety % fractional inhibitory concentrations (FIC_50_ and FIC_90_, resp.) and sum of FIC (∑FIC) were calculated as follows: FIC_50_ and FIC_90_ of drug A = IC_50_ and IC_90_ of drug A in combination/IC_50_ and IC_90_ of drug A alone. The same equation was applied to the partner drug (drug B). Sum of FIC_50_ and FIC_90_ (∑FIC_50_ and ∑FIC_90_) was calculated as follows: FIC_50_ and FIC_90_ drug A + FIC_50_ and FIC_90_ drug B. An overall mean ∑FIC_50_ and ∑FIC_90_ (*x*∑FIC_50_ and *x*∑FIC_90_) were calculated for each combination and used to classify the nature of interaction as follows: synergy (*x*∑FIC_50_ and *x*∑FIC_90_ ≤ 0.5), additivity (*x*∑FIC_50_ and *x*∑FIC_90_ between >0.5 and ≤4), and antagonism (*x*∑FIC_50_ and *x*∑FIC_90_ > 4) [13]. Isobolograms were constructed based on FIC_50_ and FIC_90_ [14] for each component of a dosage combination.

### 2.12. Statistical Analysis

The IC_50_ values were calculated using sigmoidal dose-response curves in GraphPad Prism 5.0 software. The data obtained represented the mean and standard deviation of at least two independent assays performed in duplicate or triplicate. One-way ANOVA followed by the Tukey post-test was used for significance testing (*p* < 0.05) for all assays.

## 3. Results

### 3.1. Antileishmanial and Antitrypanosomal Activity of CCBs and Cytotoxicity to Mammalian Cells

Amrinone, fendiline, lidoflazine, and mibefradil were incubated with* Leishmania* spp. promastigotes, intracellular amastigotes, and* T. cruzi *trypomastigotes to evaluate their* in vitro* antiparasitic activity. Fendiline, lidoflazine, and mibefradil showed IC_50_ values ranging from 2.75 to 16.15 *μ*M against* Leishmania *spp. promastigotes. Mibefradil was the most active compound against promastigotes, while amrinone showed lack of antileishmanial activity to the highest tested concentration of 100 *μ*M. Pentamidine was used as a standard drug in the promastigotes assay and resulted in IC_50_ values ranging from 0.69 to 1.14 *μ*M (Table 1).

The activity of fendiline and lidoflazine against intracellular amastigotes of* L. (L.) infantum* resulted in IC_50_ values ranging from 12 to 16 *μ*M. Although mibefradil was the most active compound against promastigotes, it showed lack of activity against* L. (L.) infantum *intracellular amastigotes (Table 1).

The antitrypanosomal activity was also examined and fendiline, lidoflazine, and mibefradil presented IC_50_ values, ranging from 2 to 12 *μ*M. Mibefradil was the most active drug, while amrinone was inactive. These tested compounds were considerably more effective than the standard drug benznidazole, which showed an IC_50_ of 440 *μ*M (Table 1).

In order to evaluate the cytotoxicity against mammalian cells, the tested compounds were incubated with LLC-MK2 cells, resulting in IC_50_ values ranging from 11 to 106 *μ*M (Table 1).

The Selectivity Index (SI) of tested compounds was calculated by the ratio between the cytotoxicity to LLC-MK2 cells and the antiparasitic activity. The tested compounds presented SI values ranging from 4 to 6 for* L. (L.) infantum *amastigotes and from 4 to 10 for* T. cruzi *trypomastigotes (Table 2).

### 3.2. Action of Amlodipine and Fendiline in the Mitochondrial Membrane Potential

**Figure 1:**
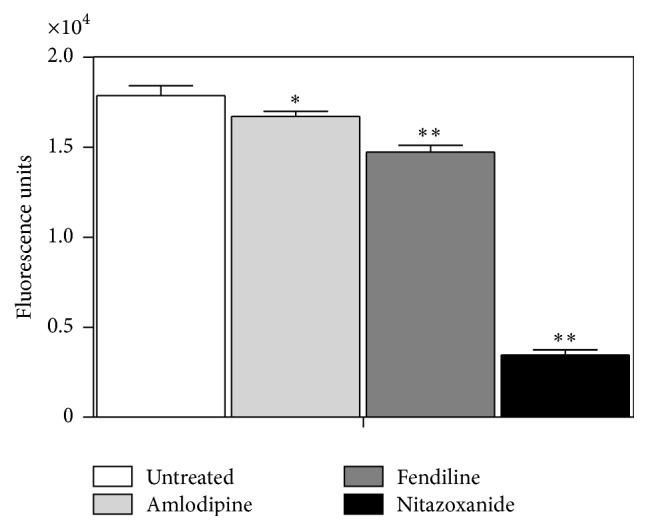
Action of amlodipine and fendiline in the mitochondrial membrane potential. Alterations in mitochondrial membrane potential were evaluated in* L. (L.) infantum* promastigotes after treatment with amlodipine and fendiline (20 *μ*M), using the fluorescent probe MitoTracker Red. Untreated parasites were used as negative control, while parasites treated with nitazoxanide were used as positive control. (*∗*) indicates significant difference relative to the untreated group (^*∗*^ *p* < 0.05; ^*∗∗*^ *p* < 0.001).

The effect of amlodipine and fendiline on the* L. (L.) infantum *mitochondrial membrane potential was evaluated in promastigotes using the fluorescent dye MitoTracker Red. Amlodipine and fendiline significantly (*p* < 0.05) affected the mitochondrial membrane potential of* L. (L.) infantum*, reducing the fluorescence levels by 7 and 18%, respectively, relative to untreated parasites. Nitazoxanide was used as positive control and resulted in a strong reduction of fluorescence intensity (Figure 1).

### 3.3. Generation of Reactive Oxygen Species (ROS) upon Treatment with Amlodipine or Fendiline

**Figure 2:**
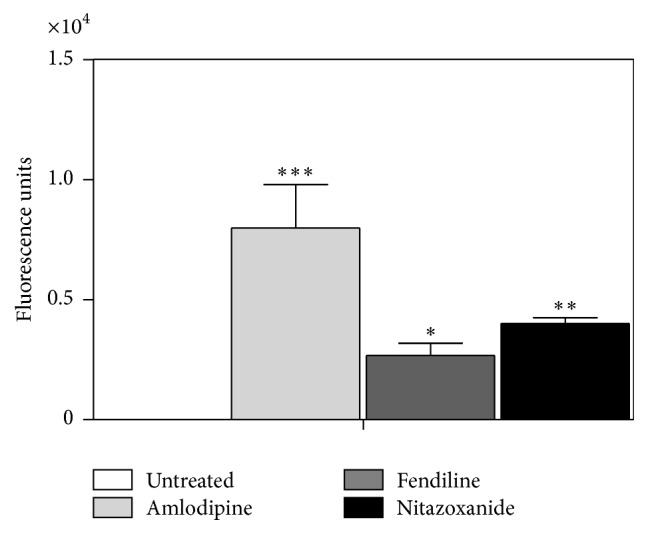
ROS generation in promastigotes in the presence of amlodipine and fendiline. The ROS production was verified using the indicator 2′,7′-dichlorodihydrofluorescein diacetate (H_2_DCF-DA) after incubation with amlodipine and fendiline (20 *μ*M). Nitazoxanide was used as positive control. (*∗*) indicates significant difference relative to the untreated group (^*∗*^ *p* < 0.05; ^*∗∗*^ *p* < 0.001; ^*∗∗∗*^ *p* < 0.001).

The regulation of ROS levels was examined using the fluorescent probe 2′,7′-dichlorodihydrofluorescein diacetate (H_2_DCF-DA) after incubation with the drugs. Amlodipine and fendiline promoted an intense and significant (*p* < 0.05) upregulation of ROS content when compared to untreated parasites (Figure 2). Amlodipine induced almost 3-fold higher ROS levels in* L. (L.) infantum *when compared to fendiline. Nitazoxanide was used as positive control and resulted in significant upregulation of ROS relative to untreated parasites, as previously reported [9]. Amlodipine presented 1.99-fold higher upregulation of ROS than nitazoxanide (Figure 2).

### 3.4. Effect of Amlodipine and Fendiline on Plasma Membrane Permeability of* L. (L.) infantum*

In order to evaluate if the mitochondrial dysfunction was a result of any alteration in plasma membrane, amlodipine and fendiline were incubated with* L. (L.) infantum *promastigotes and examined by the fluorescent probe SYTOX Green assay. According to the fluorescence intensity of untreated parasites, both drugs caused no interference on the plasma membrane permeability up to 60-minute incubation (results not shown). Untreated parasites were used as negative control and resulted in no alteration in plasma membrane permeability, while Triton X-100 was used as positive control and resulted in fully permeabilized parasites.

### 3.5. Combination of Fendiline and Antileishmanial Standard Drugs

The interactions between fendiline and three antileishmanial standard drugs (amphotericin B, miltefosine, and pentamidine) were investigated in* L. (L.) infantum* promastigotes by the modified isobologram method. In order to calculate the FIC_50_ and FIC_90_ of both drugs, the IC_50_ and IC_90_ of individual drugs were calculated and compared with the IC_50_ and IC_90_ values of each association. For each drug combination, *x*∑FIC_50_ and *x*∑FIC_90_ of both drugs were calculated, as shown in Table 3. The combination of fendiline and amphotericin B, miltefosine and pentamidine resulted in *x*∑FIC_50_ and *x*∑FIC_90_ ranging from 0.99 to 1.30, indicating indifferent/additive interaction according to the adopted classification (Table 3).

**Figure 3:**
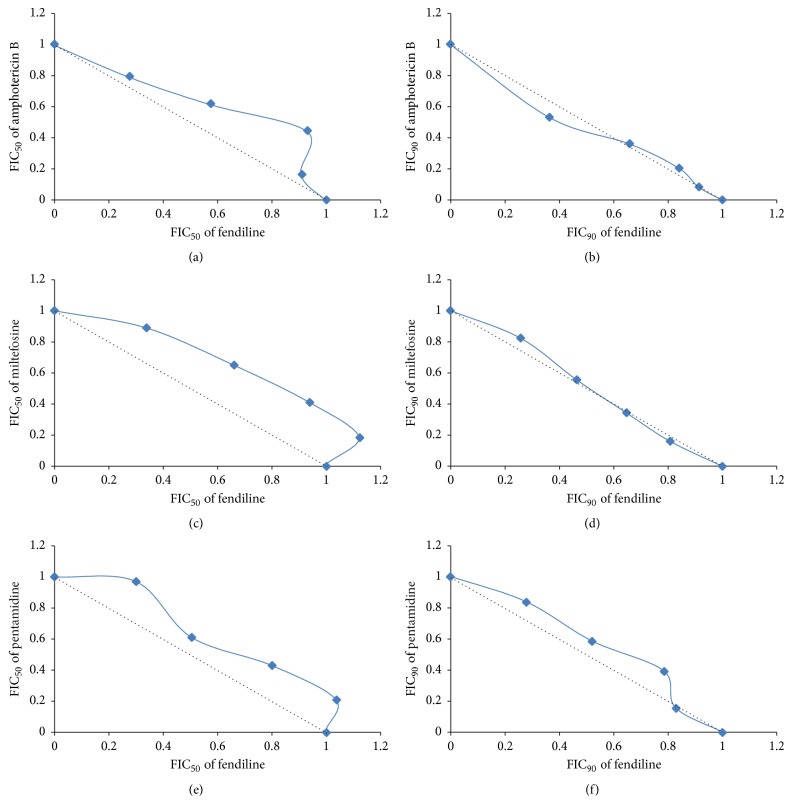
Isobolograms constructed based on the combined effect of fendiline and the antileishmanial drugs. Isobolograms generated based on FIC_50_ (left side) and FIC_90_ (right side) values showing the interaction between fendiline and antileishmanial standard drugs against* L. (L.) infantum *promastigotes. Fendiline plus amphotericin B (a and b), miltefosine (c and d), and pentamidine (e and f). The dotted line corresponds to the predicted position of the experimental points for a simple additive effect and points corresponding to the FIC values were connected by a tendency line.

In order to explore and visualize the combined effect of fendiline and the antileishmanial drugs, isobolograms were constructed based on the FIC_50_ and FIC_90_ values of each combination (Figure 3). The additive isobole was indicated by a straight dotted line intercepting the axes, when FIC = 1. Points corresponding to the FIC_50_ and FIC_90_ values of each combination were connected by a tendency line. Thus, it is noted that all points are located slightly above or below the additive isobole, close to the range that classifies these interactions as indifferent/additive.

## 4. Discussion

The search for new, safe, and cost-benefit therapies against Leishmaniasis and Chagas' disease remains a priority for developing nations. Drug repositioning or repurposing has been a successful approach for neglected diseases [15, 16].

Calcium channel blockers in clinical use for hypertension and heart diseases have been considered privileged structures, demonstrating a wide range of promising biological activities, among them,* in vivo *experimental efficacy against Ebola virus [17],* in vitro* activity against filovirus [18], and also against protozoan parasites as* Leishmania *[12],* T. cruzi *[7, 19–21], and* Plasmodium *[22, 23].

In the present work, the* in vitro *activity of fendiline and lidoflazine against* Leishmania* spp. promastigotes,* T. cruzi *trypomastigotes, and* L. (L.) infantum *intracellular amastigotes was demonstrated. Mibefradil also presented activity against* Leishmania *spp. promastigotes and* T. cruzi *trypomastigotes but was ineffective against intracellular amastigotes, probably due to metabolic differences between the extracellular and intracellular parasites or even to poor penetration of the drug into the host cells. This is the first report of antiprotozoal activity of these compounds which are non-dihydropyridine-CCBs. In a previous work, the dihydropyridines isradipine and lacidipine demonstrated* in vitro* activity against* T. cruzi *epimastigotes, with IC_50_ values of 20 and 33 *μ*M, respectively [20] showing a similar activity to fendiline and lidoflazine against* T. cruzi *trypomastigotes in the present study.

Otherwise, amrinone, a positive inotropic cardiotonic with vasodilatador properties, showed lack of activity against* Leishmania* spp. and* T. cruzi*. This result suggests that the antiparasitic activity of CCBs is rather ascribed to the chemical structure of individual compounds than to the CCB properties. Conversely, another closely related compound used as a potassium channel blocker, 4-aminopyridine, exhibited activity against* L. (L.) amazonensis*, with an IC_50_ value of 46 *μ*M, but also lacked activity against* L. (L.) major* (IC_50_ > 400 *μ*M) [24].

The mode of action of CCBs involves the blockage of calcium ions movement through calcium channels [3]. Calcium ions play an important role in regulation of many vital functions. By penetrating the cell, they activate bioenergetic processes, as the transformation of ATP into cyclic AMP and protein phosphorylation. In high concentrations, calcium ions cause different destructive changes [25].

The presence of a voltage gated calcium channel sharing several characteristics with the human counterpart has been recently demonstrated in the plasma membrane of* Leishmania *[26]; then interference of CCBs in calcium channels of treated parasites cannot be ruled out.

In a previously published work, nimodipine, a dihydropyridine, has exhibited* in vitro* activity against* L. (L.) infantum*, causing extensive mitochondrial damage in treated parasites, as observed by transmission electron microscopy [6]. In our study, fendiline demonstrated the higher potency against* Leishmania *amastigotes and it was selected for the investigation of the mechanism of action and drug combination assays. For comparisons, amlodipine, a dihydropyridine-CCB, with previously reported activity against* Leishmania* parasites [7], was included in the assays. Amlodipine presented IC_50_ values against* Leishmania* spp. close to mibefradil, but about 2- to 3-fold higher potency against* L. (L.) infantum* intracellular amastigotes than fendiline and lidoflazine.

Our data demonstrated that* Leishmania* promastigotes treated with amlodipine and fendiline exhibited reduced ability to concentrate the dye (MitoTracker Red), indicating a collapse of the mitochondrial membrane potential. This result is an indication that the energy-coupling system in the mitochondria is most likely inactivated, leading to parasite death. Another study demonstrated that* Leishmania* promastigotes treated with nimodipine also resulted in strong mitochondrial damage within 60 min incubation [7].

There is inherent relationship between ROS generation and respiratory chain in both mammals and* Leishmania. *The mitochondrial complex III was described as the main source of superoxide anion radicals [27]. Due to the observed effect of fendiline in the mitochondrial membrane potential, we also investigated the ROS levels of parasites treated with fendiline and amlodipine. We observed that depolarization in mitochondrial membrane potential was accompanied by an increase in ROS production when parasites were treated with both drugs. The single mitochondrion is one of the major sources of ROS in trypanosomatids, even under physiological conditions. These reactive species could play different roles in the parasites, involving signaling or cytotoxicity; to control the ROS levels trypanosomatids present mitochondrial antioxidant defenses [28]. The upregulation of ROS in* Leishmania* induced by fendiline and amlodipine might have contributed to a cellular toxicity, leading to an oxidative stress and parasite death.

In order to evaluate whether the fast and strong mitochondrial damage could be ascribed to the ability of amlodipine and fendiline to alter the plasma membrane of* Leishmania*, we investigated the permeability using the fluorescent probe SYTOX Green. Previous ultrastructural observation of nimodipine treated promastigotes revealed plasma membrane blebbing, although no pore forming activity could be observed [6]. In the present work, we observed that treatment with amlodipine and fendiline resulted in lack of significant changes in fluorescence intensity up to 60-minute incubation, suggesting no alteration in permeability levels. Parasites treated with Triton X-100 (positive control) showed early and increased penetration of the dye SYTOX Green and are indicative of membrane rupture.

The effects of several drugs that interfere directly with mitochondrial physiology in parasites such as* Leishmania* have been described. The unique mitochondrial features of* Leishmania* make this organelle an ideal drug target [29]. Taken together, our results demonstrate that amlodipine and fendiline exert their antileishmanial effect on* Leishmania* promastigotes due to the disruption in the mitochondrial function and to the generation of ROS.

Considering the need for new, potent, and safe treatments for Leishmaniasis, the use of monotherapy may not be the ideal future. Drug combinations are used to prevent resistance and increase safety of treatments. It has been widely studied for cancer [30], malaria, and also Leishmaniasis [31, 32]. Previous report demonstrated combinations of four dihydropyridine-CCBs (amlodipine, lercanidipine, nicardipine, and nimodipine) with antileishmanial drugs [12]. Here, we report the combination of a non-dihydropyridine drug, fendiline, with three antileishmanial clinically used drugs.

Drugs given in combination may produce effects that are similar to, higher or smaller than the effect predicted from their individual potencies [33]. Here, we observed that the effect of fendiline associated with amphotericin B, miltefosine, or pentamidine was equivalent, that is, equally effective when each drug was given separately, according to the *x*∑FIC and the isobologram graphic analysis. This behavior was similar to what was previously observed when using dihydropyridines and could be an indication that both dihydropyridine and non-dihydropyridine agents possess similar mode of action on* Leishmania*. However, additional studies are required to confirm this hypothesis.

## 5. Conclusions

The results of this work extend the investigation of CCBs as antiprotozoal agents and indicate that its leishmanicidal activity is related to mitochondria dysfunction and ROS generation. The combination of any of the drugs used did not show synergistic effects. On the contrary, all the isobolograms indicated indifferent/additive interaction. However, the drug combination assays indicated that the effect of fendiline plus amphotericin B, miltefosine, or pentamidine could be evaluated in future in animal models, since no* in vitro *antagonism was observed in any combination. Earlier, further assays must be conducted in order to verify the efficacy of fendiline in the treatment of* Leishmania* infected animals.

## Figures and Tables

**Table 1:** Effect of CCBs and standard drugs on parasites and mammalian cells.

Drug	IC_50_ (*μ*M) ± SD^a^					
	*L. (L.) infantum* promastigotes	*L. (L.) infantum* amastigotes	*L*. *(L.) amazonensis* promastigotes	*L. (V.) braziliensis* promastigotes	*T*. *cruzi* trypomastigotes	LLC-MK2 cytotoxicity
Amrinone	ne^b^	ne^b^	ne^b^	ne^b^	ne^b^	>500
Fendiline	16.15 ± 4.20	12.20 ± 1.74	8.66 ± 1.27	9.15 ± 0.78	12.13 ± 2.97	49.85 ± 8.16
Lidoflazine	17.67 ± 0.93	16.29 ± 4.45	11.54 ± 1.49	14.48 ± 1.08	10.39 ± 1.87	106.54 ± 57.99
Mibefradil	3.60 ± 0.11	ne^b^	2.23 ± 0.42	2.75 ± 0.39	2.99 ± 0.43	11.96 ± 1.03
Pentamidine	1.06 ± 0.12	nd^c^	1.14 ± 0.15	0.69 ± 0.04	nd^c^	23.48 ± 3.53
Glucantime^d^	nd^c^	30.15 ± 1.18	nd^c^	nd^c^	nd^c^	>500
Benznidazole	nd^c^	nd^c^	nd^c^	nd^c^	440.18 ± 39.14	>500

^a^IC50: 50% inhibitory concentration ± standard deviation (SD).

^b^ne: not effective.

^c^nd: not determined.

^d^Concentrations for Glucantime are expressed as *µ*g/mL, as the molecular weight is unknown.

**Table 2:** Selectivity Index (SI) of CCBs, given by the ratio between the cytotoxicity to LLC-MK2 cells and the antiparasitic activity.

Drug	*L. (L.) infantum* amastigotes	*T. cruzi* trypomastigotes
Amrinone	nd^a^	nd^a^
Fendiline	4.09	4.11
Lidoflazine	6.54	10.25
Mibefradil	nd^a^	4.01

^a^nd: not determined.

**Table 3:** Effect of combination of fendiline and antileishmanial standard drugs in *L. (L.) infantum* promastigotes.

Combination	*x*∑FIC_50_^a^	*x*∑FIC_90_^a^
Fendiline + amphotericin B	1.18	0.99
Fendiline + miltefosine	1.30	1.02
Fendiline + pentamidine	1.22	1.10

^a^Overall mean sum.
